# Rapid Determination of Per- and Polyfluoroalkyl Substances (PFAS) in Harbour Porpoise Liver Tissue by HybridSPE^®^–UPLC^®^–MS/MS

**DOI:** 10.3390/toxics9080183

**Published:** 2021-08-01

**Authors:** Simone Trimmel, Kristine Vike-Jonas, Susana V. Gonzalez, Tomasz Maciej Ciesielski, Ulf Lindstrøm, Bjørn Munro Jenssen, Alexandros G. Asimakopoulos

**Affiliations:** 1 Department of Chemistry, Norwegian University of Science and Technology (NTNU), NO-7491 Trondheim, Norway; simone.trimmel@ntnu.no (S.T.); kristine.vike@ntnu.no (K.V.-J.); susana.v.gonzalez@ntnu.no (S.V.G.); 2 Department of Biology, Norwegian University of Science and Technology (NTNU), NO-7491 Trondheim, Norway; tomasz.m.ciesielski@ntnu.no (T.M.C.); bjorn.munro.jenssen@ntnu.no (B.M.J.); 3 FRAM Centre, Institute of Marine Research, NO-9007 Tromsø, Norway; ulf.lindstroem@hi.no; 4 Department of Arctic and Marine Biology, UiT The Arctic University of Norway, NO-9037 Tromsø, Norway; 5 Department of Arctic Technology, The University Centre in Svalbard (UNIS), P.O. Box 156, NO-9171 Longyearbyen, Norway; 6 Department of Bioscience, Aarhus University, P.O. Box 358, DK-4000 Roskilde, Denmark

**Keywords:** PFAS, UPLC^®^–MS/MS, PFOS, PFOA, harbour porpoises, *Phocoena phocoena*, marine mammals, HybridSPE^®^

## Abstract

A rapid hybrid solid phase extraction (HybridSPE^®^) protocol tailored to ultra-performance liquid chromatography–electrospray ionization tandem mass spectrometry (UPLC^®^–ESI–MS/MS) analysis was developed for the determination of 15 per- and polyfluoroalkyl substances (PFAS) in liver tissue from harbour porpoises (*Phocoena phocoena*). The HybridSPE^®^ technique has been applied in trace concentration bioanalysis, but it was mainly used for liquid biological media until now. In this study, the protocol was applied on tissue matrix, and it demonstrated acceptable absolute recoveries (%) ranging from 44.4 to 89.4%. The chromatographic separation was carried out using a gradient elution program with a total run time of 4 min. The inter-day method precision ranged from 2.15 to 15.4%, and the method limits of detection (LODs) ranged from 0.003 to 0.30 ng/g wet weight (w.w.). A total of 20 liver samples were analyzed to demonstrate the applicability of the developed method in liver tissue from a wildlife species.

## 1. Introduction

Per- and polyfluoroalkyl substances (PFAS) are a versatile group of chemicals used in applications where oil and water repellence, but also high thermal and chemical stability, are required [1]. Although these unique PFAS properties render these chemicals suitable for industrial applications, they are deemed problematic regarding their environmental impacts. The first observations of PFAS toxicity were reported many decades ago, but it was only in the early 2000s when both the widespread environmental occurrence of PFAS and their associated public health effects started to be acknowledged by the scientific community [2,3,4]. This led to restrictions and gradual phasing-out of specific analogues, e.g., perfluorooctanoic acid (PFOA) and perfluorooctane sulfonate (PFOS) [5,6].

Currently, PFAS are detected in trace concentrations worldwide in biological and environmental matrices, even at remote and pristine locations [7,8,9]. To date, several bioanalytical methods are available for the determination of PFAS in a variety of biological matrices, and liquid chromatography–tandem mass spectrometry (LC-MS/MS) is currently the main instrumental technique applied for their analysis, achieving low limits of detection that can reach the picogram range [10]. Liquid (LE) and solid phase extraction (SPE) protocols are commonly used for extraction and purification purposes of biological media for PFAS determination [11]. However, a major bioanalytical drawback is the uncertainty of PFAS quantification due to matrix effects that derive from the endogenous protein and phospholipid content of samples [12]. Another important challenge of PFAS biomonitoring is the presence of background concentrations that by default hinder the measurements of trace concentrations in biological media [9,13]. In addition to this point, background PFAS contamination can derive from laboratory materials and analytical instruments during sampling and instrumental analysis, highlighting the necessity of using suitable field and procedural blanks for biomonitoring purposes [9]. Glass materials should be avoided since some analogues adsorb irreversibly to those, leading to an underestimation of actual PFAS concentrations [14].

With this background, a methodology using HybridSPE^®^ extraction tailored to UPLC^®^–ESI (electrospray)–MS/MS analysis was developed in the present study for the simultaneous determination of 15 PFAS—namely, perfluoro-n-pentanoic acid (PFPeA; C5), perfluoro-n-hexanoic acid (PFHxA; C6), perfluoro-n-heptanoic acid (PFHpA; C7), PFOA (C8), perfluoro-n-nonanoic acid (PFNA; C9), perfluoro-n-decanoic acid (PFDA; C10), perfluoro-n-undecanoic acid (PFUnA; C11), perfluoro-n-dodecanoic acid (PFDoA; C12), perfluoro-n-tridecanoic acid (PFTrA; C13), perfluorotetradecanoic acid (PFTeA; C14), perfluoro-1-butanesulfonate (PFBS; C4), perfluoro-1-hexanesulfonate (PFHxS; C6), PFOS (C8), perfluorooctanesulfonamide (PFOSA; C8), and *N*-ethylperfluoro-1-octanesulfonamide (EtFOSA; C8). In total, 20 samples of hepatic tissue from by-caught harbour porpoise (*Phocoena phocoena*) individuals were analyzed to demonstrate the applicability of the method. To our knowledge, HybridSPE^®^-based protocols are recommended by the manufacturer for biological plasma and serum and are seldom tailored to tissue applications [11].

## 2. Materials and Methods

### 2.1. Chemicals and Materials

Analytical standards of (i) perfluoroalkane sulfonates (PFSAs; 3 analogues): PFBS, PFHxS, and PFOS; (ii) perfluoroalkyl carboxylic acids (PFCAs; 10 analogues): PFPeA, PFHxA, PFHpA, PFOA, PFNA, PFDA, PFUnA, PFDoA, PFTrA, and PFTeA; and (iii) perfluoroalkane sulfonamides (FASAs; 2 analogues): EtFOSA and PFOSA; were obtained from Sigma-Aldrich (Steinheim, Germany). The isotopically labelled internal standards (ISs): perfluoro-n-octanoic acid-^13^C_8_ (PFOA-^13^C_8_, 99%) and perfluoro-1-octanesulfonate-^13^C_8_ (PFOS-^13^C_8_, 99%) were purchased from Cambridge Isotope Laboratories, Inc. (Tewksbury, MA, USA). Methanol (MeOH) of LC-MS grade and ethyl acetate (≥99.5%) were purchased from VWR Chemicals (Trondheim, Norway). Formic acid (98%), hydrochloric acid (HCl), acetic acid (≥99%), ammonium acetate (≥99%), and ammonium formate (97%) were purchased from Sigma-Aldrich (Steinheim, Germany). Water was purified with a Milli-Q grade water purification system (Q-option, Elga Labwater, Veolia Water Systems LTD, High Wycombe, UK). The SPE cartridges, HybridSPE^®^ (30 mg/1 cc), were purchased from Sigma-Aldrich (Steinheim, Germany). Metal-free polypropylene (PP; 15 and 50 mL) tubes were purchased from VWR Chemicals (Trondheim, Norway).

### 2.2. Sample Collection and Preparation

**Figure 1:**
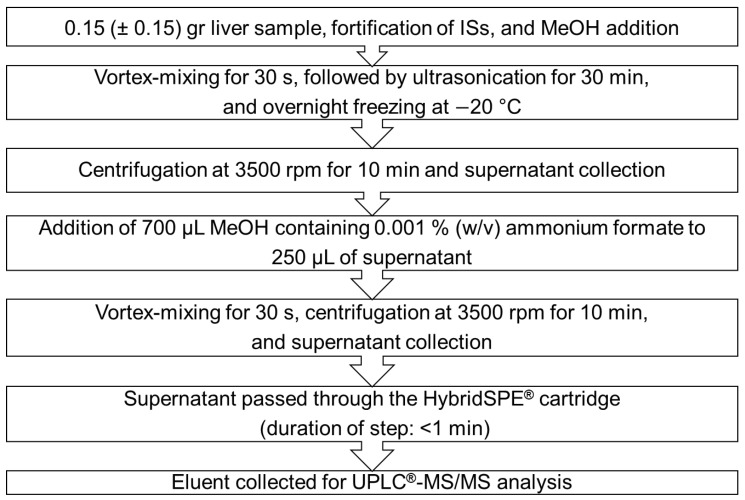
Sample preparation workflow.

Liver samples were collected from 20 harbour porpoises by-caught in gillnets along the Norwegian coast in September–October 2016 and February–April 2017 [15]. All weighed tissues were homogenized and transferred to PP (50 mL) tubes and stored in darkness at −20 °C until sample extraction. The sample preparation workflow is presented in Figure 1. A portion of 0.15 (± 0.15) g from each homogenized harbour porpoise liver was placed into a 15 mL PP tube, 10 ng of each IS was added followed by 500 μL MeOH. The samples were vortex-mixed for 30 s, ultrasonicated for 30 min, and were left overnight in the freezer at −20 °C. Thereafter, the samples were centrifuged at 3500 rpm for 10 min. A portion of 250 µL of the supernatants was transferred into new 15 mL PP tubes, and 700 µL MeOH containing 0.001 % (*w*/*v*) ammonium formate was added. The mixtures were vortex-mixed for 30 s, centrifuged at 3500 rpm for 10 min, and the supernatants were collected and passed directly through the HybridSPE^®^ cartridges. The extracts were collected and transferred directly for UPLC^®^–MS/MS analysis. For the method development and validation, matrix standards were prepared from harbour porpoise liver (pool matrix from six randomly selected samples).

### 2.3. UPLC^®^–MS/MS Analysis

The chromatographic separation was carried out using an Acquity UPLC^®^ I-Class system (Waters, Milford, CT, USA) coupled to a triple quadrupole mass analyser (QqQ; Xevo TQ-S) with a *Z*Spray ESI ion source (Waters, Milford, CT, USA). The used LC column was a Kinetex C18 (30 × 2.1 mm, 1.3 µm) connected to a Phenomenex C18 guard column (2.0 × 2.1 mm). The column temperature was set at 30 °C. The chromatographic separation was carried out using a gradient elution program with 2 mM ammonium acetate in water (A) and ΜeOH (B) as binary mobile phase with a flow rate of 0.25 µL/min. The gradient elution started at 90% A, held for 0.2 min, decreased to 0% within 2.8 min (minute 3.0), held for 0.5 min (minute 3.5), and reverted to 90% at the minute 3.6, which was held for 0.4 min, for a total run time of 4.0 min. The injection volume was 4 µL. The electrospray ionisation (ESI) was applied at a potential of −1.8 kV. The cone and source offset voltages were set at 30 and 40 V, respectively. The desolvation and cone gas flow rates were set at 900 and 150 L/h, respectively. The collision gas flow was set at 0.15 mL/min, and the nebulizer gas pressure was set at 87 psi. The source and desolvation temperatures were set at 150 and 450 °C, respectively. The method limits of detection (LODs) and quantification (LOQs) were calculated for each target analyte as 3 and 10 times the signal from the baseline noise (S/N ratio) adjusted for extraction losses and matrix effects from liver [15,16]. The LODs and LOQs of the target analytes are presented in Table 1. More details concerning the instrumental parameters of the UPLC^®^–MS/MS analysis are available in Tables S1 and S2. Concentrations are reported as ng/g wet weight (w.w.).

### 2.4. Method Validation and Data Analysis

The calibration of the ESI method was verified by injecting solvent calibration standards at concentrations of 0.05–50.0 ng/mL (0.05, 0.10, 0.20, 0.50, 1.00, 2.00, 5.00, 10.0, 20.0, 50.0 ng/mL). Quantification of the target analytes was accomplished based on the internal standard method and with matrix-matched calibration standards prepared by spiking target analytes into the liver pool matrix prior to extraction [17]. Precision was assessed through reproducibility experiments; the liver pool matrix was fortified at two amounts (10 and 20 ng) and four replicate analyses (*N* = 4) were prepared for each amount. The accuracy (trueness) was evaluated through recovery experiments at the fortified amount of 10 ng of the target PFAS; absolute and relative recoveries percentages (as defined by Asimakopoulos et al. [18]) were calculated in four replicates (*N* = 4). The method matrix effects (MEs%) for PFAS analysis were assessed at the same fortification amount in four replicates (10 ng; *N* = 4). The ion ratio (%) for every target analyte was calculated, except for PFPeA (since it lacked a confirmation ion for UPLC^®^–MS/MS analysis), from the replicates (*N* = 5) performed in standard solvent solution (10 ng/mL), as previously described [18].

### 2.5. Data Analysis and Statistical Treatment

UPLC^®^–MS/MS data were acquired with MassLynx v4.1 software, and quantification processing was performed with TargetLynx (Waters, Milford, CT, USA). Excel (Microsoft, 2018) was used for general descriptive statistics. Data analysis did not include censored data (i.e., non-detects; NDs).

## 3. Results and Discussion

### 3.1. HybridSPE^®^ Extraction

**Figure 2:**
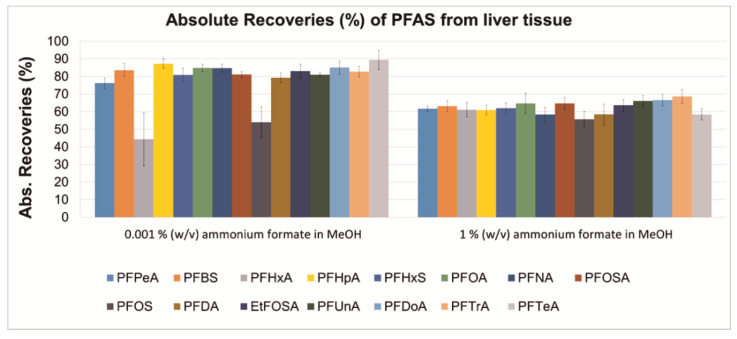
Absolute recoveries (%; *N* = 4) at the fortification amount of 10 ng on pooled harbour porpoise liver tissue with two different concentrations of ammonium formate in MeOH.

**Figure 3:**
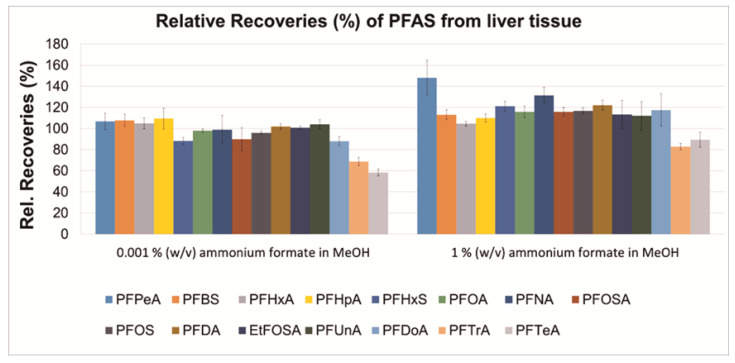
Relative recoveries (%; *N* = 4) at the fortification amount of 10 ng on pooled harbour porpoise liver tissue with two different concentrations of ammonium formate in MeOH.

**Figure 4:**
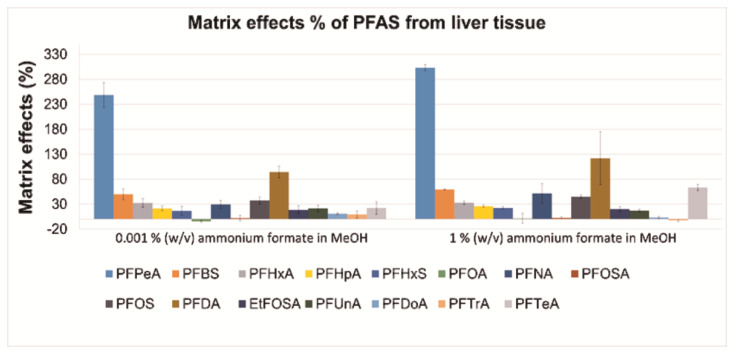
Matrix effects (%; *N* = 4) at the fortification amount of 10 ng on pooled harbour porpoise liver tissue with two different concentrations of ammonium formate in MeOH.

Two methanol-based precipitation agents, containing either 0.001 or 1 % (*w*/*v*) ammonium formate, were tested by applying the sample preparation procedure presented in Figure 1 (Section 2.2). For each precipitating agent, absolute (Figure 2) and relative recoveries (%) (Figure 3), and MEs percentages (Figure 4) are presented. The absolute recoveries percentages ranged from 44.4 to 89.4% and from 55.6 to 68.6%, with 0.001 and 1% (*w*/*v*) ammonium formate, respectively; the results indicated acceptable extraction efficiency. The relative recoveries percentages ranged from 58.4 to 109.6% and from 82.8 to 148.1%, with 0.001 and 1% (*w*/*v*) ammonium formate, respectively.

The matrix effects (MEs) percentages ranged from −4.4 (PFOA) to 248.4% (PFPeA) and from −2.3 (PFTrA) to 303.2% (PFPeA), with 0.001 and 1% (*w/v*) ammonium formate, respectively, indicating strong matrix enhancement during ESI for most target analytes; such strong matrix effects during PFAS analysis were also previously documented in literature [10]. The matrix effects were slightly stronger for all target analytes when 1% (*w*/*v*) ammonium formate in MeOH was used as precipitation agent. Overall, it was concluded that the two tested percentages of ammonium formate could be equally used in the precipitation agent. It is noteworthy that further method development/validation and analysis of samples was performed with 0.001% (*w*/*v*) ammonium formate in MeOH (absolute and relative recoveries percentages are presented numerically for this precipitation agent in Table S3).

### 3.2. UPLC^®^-MS/MS Method Performance

**Figure 5:**
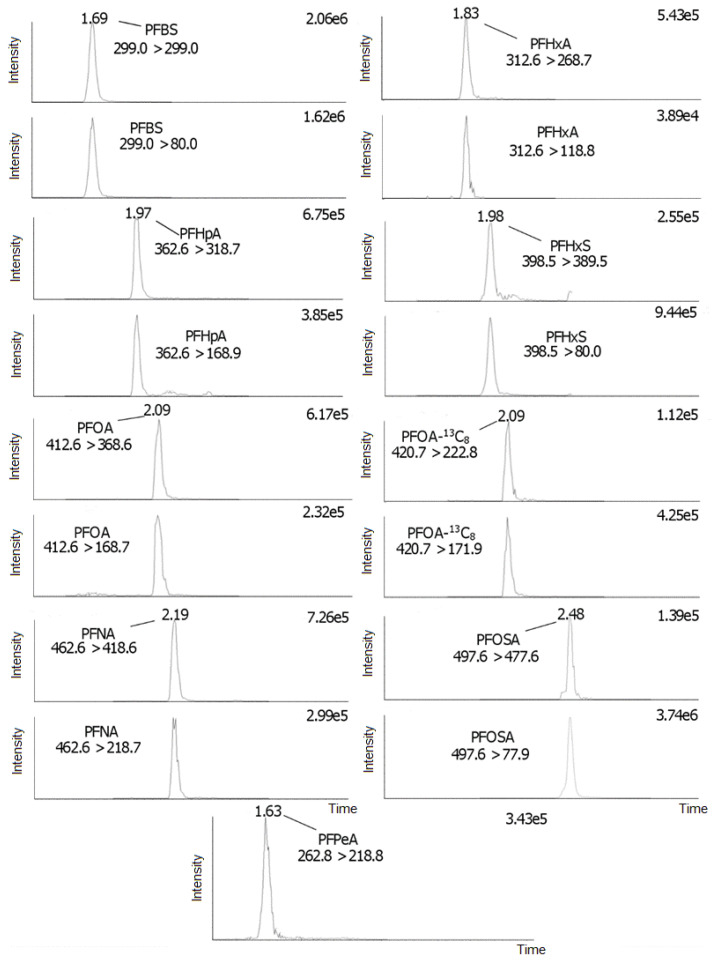
SRM ion chromatograms of PFBS, PFHxA, PFHpA, PFHxS, PFOA, PFOA-^13^C_8_, PFNA, PFOSA, and PFPeA.

**Figure 6:**
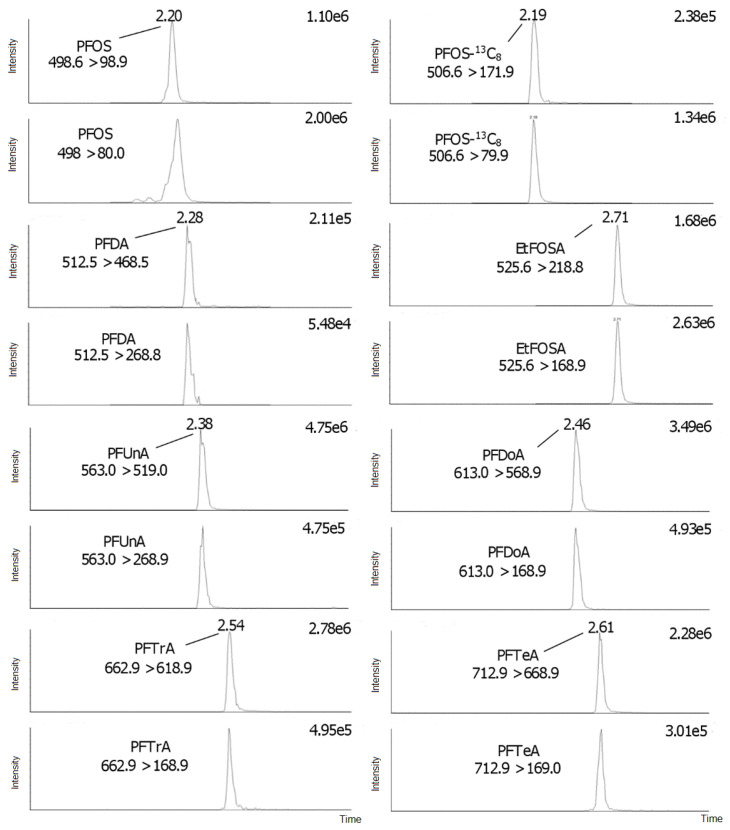
SRM ion chromatograms of PFOS, PFOS-^13^C_8_, PFDA, EtFOSA, PFUnA, PFDoA, PFTrA, and PFTeA.

The instrumental correlation coefficients for all PFAS were acceptable in the investigated intervals (*r* > 0.990). The LODs ranged from 0.003 to 0.30 ng/g w.w., and the LOQs ranged from 0.01 to 0.99 ng/g w.w. (Table 1); the values were found in the same order of magnitude as those previously reported in the literature [19,20]. The inter-day method precision (method reproducibility, RSD %, *N* = 4, k = 2 days), when calculated based on the external standard method, were 1.71–16.4% and 1.92–14.5% at the fortified amount of 10 (medium level) and 20 (high level) ng, respectively (Table 2 and Table 3). The inter-day method precision (method reproducibility, RSD %, *N* = 4, k = 2 days), when calculated based on the internal standard method, were 2.15–15.4% and 2.94–10.5% at the fortified amount of 10 (medium level) and 20 (high level) ng, respectively (Table 2 and Table 3). The method precision results showed acceptable values for all target PFAS. Overall, the low uncertainty (low RSDs %) of the recoveries with the HybridSPE^®^ protocol was attributed to the obtained visually clean extracts, but also to the lack of an evaporation and reconstitution step during sample preparation, which both steps are commonly performed in SPE- and LE-based protocols [11]. The calculated ion ratios percentages are presented in Table S1 and ranged from 6.3 to 172%. Retention (RT) and relative retention (RRT) times were 1.63–2.71 min and 0.78–1.24, respectively, demonstrating the rapidity of the UPLC^®^ instrumental method. Typical selected reaction monitoring (SRM) ion chromatograms from fortified amounts of 10 ng on liver pool matrix are presented in Figure 5 and Figure 6.

### 3.3. Method Application

A total of 20 porpoise liver samples were analysed, and the results are presented in Table 4. The highest detection rates were found for PFOS and PFOSA with 100%, followed by PFDA and PFUnA with 95%, and PFNA with 90%. The rank order of median concentrations for the most detected target analytes in the liver was: PFOS (60.1 ng/g w.w.) > PFUnA (3.03) > PFDA (2.02) > PFNA (0.76) > PFOSA (0.53). It should be noted that concentrations of PFAS differ among species, and spatially within species, due to differences in dietary composition (e.g., trophic position) and potential differences in biotransformation/elimination capacities. In general, the orders of magnitude of the concentrations found here agree with those reported in previous studies. Indicatively, in liver samples from harbour porpoises (from Western Iceland), the concentrations of PFOS, PFUnA, PFNA, and PFDA were reported ranging from 38 to 67, from 16 to 24, from 0.4 to 1.9, and from 4.1 to 4.5 ng/g w.w., respectively [20]. The occurrence of PFUnA in liver samples from white-sided dolphins (*Lagenorhynchus acutus*; from the Faroe Islands) ranged from 45 to 68 ng/g w.w. [20], and from harbour seals (*Phoca vitulina*; from Northern Norway) an average concentration of 6.88 ng/g w.w. was reported [21]. In liver samples from ringed seals (*Phoca hispida*) and minke whales (*Balaenoptera acutorostrata*), the PFOSA concentrations ranged up to 29 ng/g w.w. [22].

In this study, PFDoA and PFTrA individually demonstrated a detection rate of 70%, with a median concentration of 0.70 and 1.60 ng/g w.w., respectively. The order of magnitude of PFDoA and PFTrA concentrations agree with those found earlier in liver samples from minke and fin whales (*Balaenoptera physalus*) since these concentrations were ranging from 0.1 to 1.4 and from 0.3 to 0.7 ng/g w.w., respectively [20]. For harbour porpoises, the liver concentrations reported in the literature for PFDoA and PFTrA, although being on the same order of magnitude, they were significantly higher in absolute values from those reported here, ranging from 3.4 to 3.5 and from 4.2 to 4.9 ng/g w.w., respectively [20]. In this study, the lowest detection rates were found for PFTeA and PFHxS, with 25%, followed by PFPeA, PFHxA, PFHpA, PFOA, PFBS, and EtFOSA with 5% (detected only in one sample). The concentrations of both PFTeA and PFHxS were determined < 1 ng/g w.w., which also agreed with the previous literature on vertebrates [20,21].

## 4. Conclusions

The HybridSPE^®^ technique has so far been applied in a limited number of trace concentration bioanalytical applications, mainly for liquid biological media, and it is not commonly reported in the literature as alternative for SPE and LE. A rapid HybridSPE^®^ protocol tailored to UPLC^®^–MS/MS analysis was developed for the determination of 15 PFAS in liver from harbour porpoises. The absolute and relative recoveries (%) ranged from 44.4 to 89.4% and from 58.4 to 109.6%, respectively, with good precision (RSD: 2.15–15.4%, when the internal standard method was used). The total UPLC^®^ run time was 4.0 minutes, showing the rapidity of the instrumental method. The method was applied successfully in 20 liver tissue samples from harbour porpoises, demonstrating the highly selective and sensitive analysis of PFAS with the HybridSPE^®^ technique. The developed method validates the potential of the HybridSPE^®^ technique for use in solid tissue bioanalytical applications and allows for further in-depth studies on the presence and role of PFAS in wildlife species.

## Figures and Tables

**Table 1:** Limits of detection and quantification for the target analytes.

Target Analytes	LOD (ng/g w.w.)	LOQ (ng/g w.w.)
PFPeA	0.30	0.99
PFHxA	0.20	0.67
PFHpA	0.12	0.39
PFOA	0.09	0.29
PFNA	0.04	0.13
PFDA	0.07	0.22
PFUnA	0.03	0.09
PFDoA	0.08	0.25
PFTrA	0.12	0.41
PFTeA	0.11	0.35
PFBS	0.04	0.12
PFHxS	0.01	0.04
PFOS	0.02	0.08
PFOSA	0.003	0.01
EtFOSA	0.01	0.04

**Table 2:** Inter-day method precision (reproducibility; fortification amounts: 10 and 20 ng, RSD %, *N* = 4, k = 2 days) for PFBS, PFHxS, PFOS, PFPeA, PFHxA, PFHpA, and PFOA, presented based on the external and internal standard method.

Reproducibility	PFBS	PFHxS	PFOS	PFPeA	PFHxA	PFHpA	PFOA
10 ng (external)	4.38	4.87	16.1	4.00	16.4	3.27	2.58
10 ng (internal)	3.54	2.15	5.93	8.39	15.4	4.14	3.56
20 ng (external)	2.07	2.00	9.71	4.82	14.5	2.07	1.92
20 ng (internal)	5.25	5.19	3.94	7.00	10.5	4.63	4.81

**Table 3:** Inter-day method precision (reproducibility; fortification amounts: 10 and 20 ng, RSD %, *N* = 4, k = 2 days) for PFNA, PFDA, PFUnA, PFDoA, PFTrA, PFTeA, EtFOSA, and PFOSA, presented based on the external and internal standard method.

Reproducibility	PFNA	PFDA	PFUnA	PFDoA	PFTrA	PFTeA	EtFOSA	PFOSA
10 ng (external)	2.79	3.43	1.71	4.37	3.59	6.19	4.73	2.16
10 ng (internal)	3.94	3.45	3.91	11.7	12.0	13.0	2.42	4.63
20 ng (external)	3.21	2.62	3.07	3.03	2.93	3.53	2.10	2.37
20 ng (internal)	3.98	3.46	4.45	4.09	3.89	4.25	2.96	2.94

**Table 4:** Concentration and detection rates of PFAS in harbour porpoise liver (*N* = 20).

Target Analytes	Mean (ng/g w.w.)	Median (ng/g w.w.)	Min (ng/g w.w.)	Max (ng/g w.w.)	Detection Rate (%)
PFPeA	46.7	46.7	46.7	46.7	5 *
PFHxA	159	159	159	159	5 *
PFHpA	1.12	1.12	1.12	1.12	5 *
PFOA	0.71	0.71	0.71	0.71	5 *
PFNA	0.80	0.76	0.32	2.12	90
PFDA	3.03	2.02	0.51	12.7	95
PFUnA	4.94	3.03	0.94	16.7	95
PFDoA	0.93	0.69	0.29	2.31	70
PFTrA	1.81	1.62	0.50	4.32	70
PFTeA	0.49	0.50	0.33	0.77	25
PFBS	0.48	0.48	0.48	0.48	5 *
PFHxS	0.88	0.75	0.51	1.50	25
PFOS	69.2	60.1	2.63	194	100
PFOSA	0.61	0.53	0.20	1.11	100
EtFOSA	0.42	0.42	0.42	0.42	5 *

* Detected only in one sample (out of the 20).

## Data Availability

Data are contained within the article or Supplementary Materials.

## Supplementary Materials

The following are available online at https://www.mdpi.com/article/10.3390/toxics9080183/s1, Table S1: SRM (Selected Reaction Monitoring) transitions, collision energies, and cone voltage values for the UPLC^®^–MS/MS analysis of PFAS, Table S2: Retention (RT) and relative retention (RRT) times of the UPLC^®^–MS/MS method, and presenting the internal standard (IS) used for the quantification of every target analyte, Table S3: Mean absolute and relative recoveries (%) of the target analytes at a fortified amount of 10 ng with 0.001% (*w*/*v*) ammonium formate in methanol as the precipitation agent.

## References

[1] Lemal D.M. (2004). Perspective on Fluorocarbon Chemistry. J. Org. Chem..

[2] Nordby G.L., Luck J.M. (1955). Perfluorooctanoic acid interactions with human serum albumin. J. Biol. Chem..

[3] Lewis C.E., Kerby G.R. (1965). An Epidemic of Polymer-Fume Fever. J. Am. Med. Assoc..

[4] Renner R. (2001). Growing Concern Over Perfluorinated Chemicals. Environ. Sci. Technol..

[5] (2006). Directive 2006/122/EC of the European Parliament and of the Council of 12 December 2006. Amending for the 30th time Council Directive 76/769/EEC on the Approximation of the Laws, Regulations and Administrative Provisions of the Member States Relating to r. https://eur-lex.europa.eu/legal-content/EN/TXT/PDF/?uri=CELEX:32006L0122&from=EN.

[6] (2020). EU Comission 2020/784 Commission Delegated Regulation (EU) 2020/784 of 8 April 2020 Amending Annex I to Regulation (EU) 2019/1021 of the European Parliament and of the Council as Regards the Listing of Perfluorooctanoic Acid (PFOA), Its Salts and PFOA-Related Compounds. https://eur-lex.europa.eu/legal-content/EN/TXT/HTML/?uri=CELEX:32020R0784&from=EN.

[7] Ask A.V., Jenssen B.M., Tartu S., Angelier F., Chastel O., Gabrielsen G.W. (2021). Per- and Polyfluoroalkyl Substances Are Positively Associated with Thyroid Hormones in an Arctic Seabird. Environ. Toxicol. Chem..

[8] Joerss H., Xie Z., Wagner C.C., Von Appen W.-J., Sunderland E.M., Ebinghaus R. (2020). Transport of Legacy Perfluoroalkyl Substances and the Replacement Compound HFPO-DA through the Atlantic Gateway to the Arctic Ocean-Is the Arctic a Sink or a Source. Environ. Sci. Technol..

[9] Taniyasu S., Kannan K., So M.K., Gulkowska A., Sinclair E., Okazawa T., Yamashita N. (2005). Analysis of fluorotelomer alcohols, fluorotelomer acids, and short- and long-chain perfluorinated acids in water and biota. J. Chromatogr. A.

[10] Arvaniti O.S., Asimakopoulos A.G., Dasenaki M.E., Ventouri E.I., Stasinakis A.S., Thomaidis N.S. (2014). Simultaneous determination of eighteen perfluorinated compounds in dissolved and particulate phases of wastewater, and in sewage sludge by liquid chromatography-tandem mass spectrometry. Anal. Methods.

[11] Honda M., Robinson M., Kannan K. (2018). A rapid method for the analysis of perfluorinated alkyl substances in serum by hybrid solid-phase extraction. Environ. Chem..

[12] Ahmad S., Kalra H., Gupta A., Raut B., Hussain A., Rahman M.A. (2012). HybridSPE: A novel technique to reduce phospholipid-based matrix effect in LC-ESI-MS Bioanalysis. J. Pharm. Bioallied Sci..

[13] Bartlett S.A., Davis K.L. (2018). Evaluating PFAS cross contamination issues. Remediation.

[14] Martin J.W., Kannan K., Berger U., De Voogt P., Field J., Franklin J., Giesy J.P., Harner T., Muir D.C.G., Scott B. (2004). Analytical challenges hamper perfluoroalkyl research. Environ. Sci. Technol..

[15] Rian M.B., Vike-Jonas K., Gonzalez S.V., Ciesielski T.M., Venkatraman V., Lindstrøm U., Jenssen B.M., Asimakopoulos A.G. (2020). Phthalate metabolites in harbor porpoises (*Phocoena phocoena*) from Norwegian coastal waters. Environ. Int..

[16] Vike-Jonas K., Gonzalez S.V., Mortensen Å.K., Ciesielski T.M., Farkas J., Venkatraman V., Pastukhov M.V., Jenssen B.M., Asimakopoulos A.G. (2021). Rapid determination of thyroid hormones in blood plasma from Glaucous gulls and Baikal seals by HybridSPE®-LC-MS/MS. J. Chromatogr. B Anal. Technol. Biomed. Life Sci..

[17] Asimakopoulos A.G., Xue J., De Carvalho B.P., Iyer A., Abualnaja K.O., Yaghmoor S.S., Kumosani T.A., Kannan K. (2016). Urinary biomarkers of exposure to 57 xenobiotics and its association with oxidative stress in a population in Jeddah, Saudi Arabia. Environ. Res..

[18] Asimakopoulos A.G., Thomaidis N.S. (2015). Bisphenol A, 4-t-octylphenol, and 4-nonylphenol determination in serum by Hybrid Solid Phase Extraction-Precipitation Technology technique tailored to liquid chromatography-tandem mass spectrometry. J. Chromatogr. B Anal. Technol. Biomed. Life Sci..

[19] Kelly B.C., Ikonomou M.G., Blair J.D., Surridge B., Hoover D., Grace R., Gobas F.A.P.C. (2009). Perfluoroalkyl Contaminants in an Arctic Marine Food Web: Trophic Magnification and Wildlife Exposure. Environ. Sci. Technol..

[20] Rotander A., Kärrman A., Van Bavel B., Polder A., Rigét F., Auðunsson G.A., Vikingsson G., Gabrielsen G.W., Bloch D., Dam M. (2012). Increasing levels of longchain perfluorocarboxylic acids (PFCAs) in Arctic and North Atlantic marine mammals, 1984–2009. Chemosphere.

[21] NILU (2013). Perfluorinated Alkylated Substances (PFAS), Brominated Flame Retardants (BFR) and Chlorinated Paraffins (CP) in the Norwegian Environment—Screening.

[22] Bossi R., Riget F.F., Dietz R., Sonne C., Fauser P., Dam M., Vorkamp K. (2005). Preliminary screening of perfluorooctane sulfonate (PFOS) and other fluorochemicals in fish, birds and marine mammals from Greenland and the Faroe Islands. Environ. Pollut..

