# Supplementary material for: Rapid Determination of Per- and Polyfluoroalkyl Substances (PFAS) in Harbour Porpoise Liver Tissue by HybridSPE®–UPLC®–MS/MS
Source: Toxics. 2021 Aug 1;9(8):183. doi: 10.3390/toxics9080183 (PMC8402602; doi:10.3390/toxics9080183)
Supplement: Supplementary file 1 [file toxics-09-00183-s001.zip › toxics-1289821-supplementary.pdf]

toxics MDPI

Supplementary Materials: Rapid Determination of Per- and Polyfluoroalkyl Substances (PFAS) in Harbour Porpoise Liver Tissue by HybridSPE®-UPLC®-MS/MS

Simone Trimmel, Kristine Vike-Jonas, Susana V. Gonzalez, Tomasz Maciej Ciesielski, Ulf Lindstrøm, Bjørn Munro Jenssen and Alexandros G. Asimakopoulos

Table S1. SRM (Selected Reaction Monitoring) transitions, collision energies, and cone voltage values for the UPLC®-MS/MS analysis of PFAS.

Target Analytes	Transition 1*	Transition 2	Collision energy (V)	Cone voltage values (V)	Ion ratio %#
PFBS	299>80	299>299	26; 24	22; 8	172
PFHxS	399>80	399>399	34; 34	50; 8	69.8
PFOS	499>99	499>80	44; 44	56; 36	89.8
PFPeA	263>219	-	8	20	-
PFHxA	313>269	313>119	8;18	10;10	4.20
PFHpA	363>169	363>319	16;10	16;16	35.0
PFOA	413>369	413>169	8;18	20;20	27.1
PFNA	463>419	463>219	10;16	20;20	19.1
PFDA	513>469	513>269	10;18	16;16	11.1
PFUnA	563>519	563>269	10;18	12;12	12.7
PFDoA	613>569	613>169	12;26	26;26	12.1
PFTrA	663>619	663>169	12;24	28;28	12.9
PFTeA	713>669	713>169	14;30	20;20	13.9
PFOSA	498>78	498>478	28;28	12;12	6.3
EtFOSA	526>169	526>219	44;44	26;26	69.6
PFOA-C^{13}	421>172	421>223	16;16	16;16	-
PFOS-C^{13}	507>80	507>172	56;56	46;32	-

* Ion transition with higher signal intensity (used as the quantification ion).
# Calculated from the replicates (*N* = 5) performed in a standard solvent solution (10 ng/mL).

Table S2. Retention (RT) and relative retention (RRT) times of the UPLC®-MS/MS method, and presenting the internal standard (IS) used for the quantification of every target analyte.

Target Analytes	RT (min)	RRT	IS
PFBS	1.69	0.80	PFOA-^{13}C
PFHxS	1.97	0.95	PFOA-^{13}C
PFOS	2.20	1.00	PFOS-^{13}C
PFPeA	1.63	0.78	PFOA-^{13}C
PFHxA	1.83	0.88	PFOA-^{13}C
PFHpA	1.98	0.95	PFOA-^{13}C
PFOA	2.09	1.00	PFOA-^{13}C
PFNA	2.19	1.00	PFOS-^{13}C
PFDA	2.28	1.04	PFOS-^{13}C
PFUnA	2.38	1.08	PFOS-^{13}C
PFDoA	2.46	1.12	PFOS-^{13}C
PFTrA	2.54	1.16	PFOS-^{13}C
PFTeA	2.61	1.19	PFOS-^{13}C
PFOSA	2.48	1.11	PFOS-^{13}C
EtFOSA	2.71	1.24	PFOS-^{13}C
PFOA-^{13}C	2.09	-	-
PFOS-^{13}C	2.19	-	-

Table S3. Mean absolute and relative recoveries (%; *N*=4) of the target analytes at a fortified amount of 10 ng with 0.001 % (*w*/*v*) ammonium formate in methanol as the precipitation agent.

**Target analyte [IS used for the calculation of the relative recovery (%)]**	**Absolute recovery (%)**	**Relative recovery (%)**
PFPA (PFOA-^{13}C)	76.2	98.7
PFHxA (PFOA-^{13}C)	44.4	148.1
PFHpA (PFOA-^{13}C)	87.3	113.1
PFOA (PFOA-^{13}C)	84.8	109.9
PFNA (PFOS-^{13}C)	84.7	121.1
PFDA (PFOS-^{13}C)	79.3	115.9
PFUnA (PFOS-^{13}C)	81.0	122.0
PFDoA (PFOS-^{13}C)	85.0	113.2
PFTrA (PFOS-^{13}C)	82.8	112.0
PFTeA (PFOS-^{13}C)	89.4	117.5
PFBS (PFOA-^{13}C)	83.6	108.2
PFHxS (PFOA-^{13}C)	80.9	104.6
PFOS (PFOS-^{13}C)	54.0	131.2
PFOSA (PFOS-^{13}C)	81.1	115.8
EtFOSA (PFOS-^{13}C)	83.0	116.8
